# Multiple 11C-acetate and 18fludeoxyglucose-avid hepatic tumours in a young woman: primary, secondary, or something else?

**DOI:** 10.1093/jscr/rjaf933

**Published:** 2025-11-27

**Authors:** Kin Pan Au, Regina Lo, Kenneth S H Chok

**Affiliations:** Division of Hepatobiliary and Pancreatic Surgery, Department of Surgery, Queen Mary Hospital, The University of Hong Kong, Pokfulam Road, HK, Hong Kong; Department of Pathology, Queen Mary Hospital, The University of Hong Kong, Pokfulam Road, HK, Hong Kong; Department of Surgery, Faculty of Medicine, The Chinese University of Hong Kong, Prince of Wales Hospital, Shatin, N.T. Hong Kong

**Keywords:** hepatic adenoma, liver tumour, PET-CT

## Abstract

A 32-year-old lady presented with abdominal pain and computed tomography scan detected multifocal bilobar hepatic tumours. The tumours had heterogeneous radiological appearance. The largest tumour situated in left lateral section and was hypodense on arterial and portal venous phases. There were two small arterial enhancing tumours at segment 4a and 8, respectively. On dual-tracer positron emission tomography scan, these tumours were mostly 11C-acetate and 18FDG-avid, radiologically mimicking multifocal hepatocellular carcinoma. Immuno-histochemical staining after resection confirmed the diagnosis of inflammatory hepatic adenoma. The tumour cells expressed serum amyloid A and C-reactive protein.

## Introduction

Hepatocellular adenoma (HCA) is a potentially premalignant tumour arising from hepatocytes. It is associated with oestrogen exposure and is frequently diagnosed in young women with oral contraceptive use [1, 2]. Recent advances in molecular genetics have redefined HCA into subtypes with distinct clinical features (Table 1). We hereby describe a case of multiple inflammatory HCA with 11C-acetate and 18fludeoxyglucose (FDG) avidity on dual tracer positron emission tomography (PET) scan, mimicking multifocal hepatocellular carcinoma (HCC).

**Table 1:** Molecular classification of HCA

Genetic alteration	Clinical	Radiological	Pathological
HNF-1α inactivation	Familial liver adenomatosis	T1 signal dropout	Steatosis
β-catenin activating at exon 3	Malignant transformation		Cellular atypia
β-catenin activating at exon 7 and 8			
Inflammatory	Obesity/alcohol	T2 hyperintensity	Telangiectasia
Sonic hedgehog	Tumour bleeding		Heamorrhagic foci
Unclassified			

## Case report

**Figure 1:**
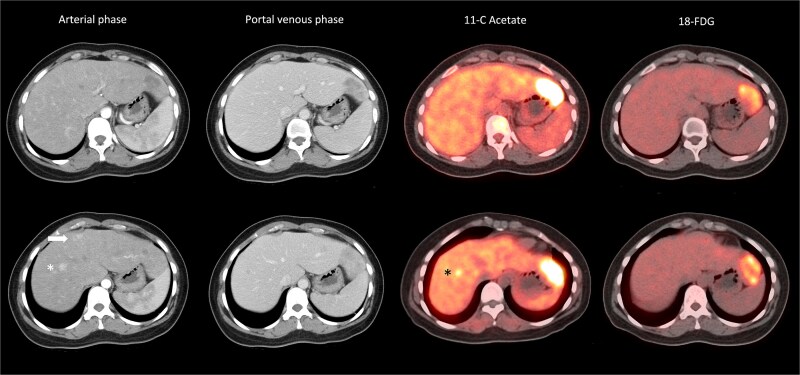
Heterogeneous radiological appearance of the tumours. Lateral S2 tumour was hypodense on arterial phase, and with ^11^C-acetate and ^18^FDG avidity; S4a tumour (arrow) was arterial enhancing but nonavid on PET. S8 lesion (^*^) was arterial enhancing and with ^11^C-acetate but not ^18^FDG avidity.

A 32-year-old lady with good past health presented with abdominal pain and computed tomography (CT) scan detected six tumours over both lobes of the liver (Table 2). She was not a chronic hepatitis carrier and had taken oral contraceptives for several months prior to presentation. The tumours had heterogeneous radiological appearance (Fig. 1). Four of the tumours were situated in the left lateral section. The largest measured 6 cm in diameter and was hypodense on arterial and portal venous phases. There were two small arterial enhancing tumours at segment 4a and 8, respectively. They were no portal venous washout. Serum level of alpha-fetoprotein was normal. On dual-tracer PET scan, these tumours were mostly 11C-acetate and 18FDG-avid (Fig. 1). In the view of inconclusive radiological findings, percutaneous biopsy of the largest tumour at lateral S2 was performed, showing regenerating hepatocytes with marked macro-vesicular steatosis affecting 80% of the tissue core. Pathological findings suggested an HCA.

**Table 2:** Radiological features of the tumours

	Size (cm)	Arterial	Portal venous	11C-acetate	18-FDG
Superior S2	1.6 × 1.6	Enhancing	Isodense	6.1	3.9
Lateral S2	6.0 × 2.4	Hypodense	Hypodense	9.2	7.0
Medial S2	1.7 × 1.7	Enhancing	Isodense	9.2	6.6
S3	4.5 × 2.6	Hypodense	Hypodense	10.1	7.8
S4a	1.0 × 1.0	Enhancing	Isodense		
S8	1.2 × 1.2	Enhancing	Isodense	7.0	
Background liver SUVmax:				5.5	2.7

**Figure 2:**
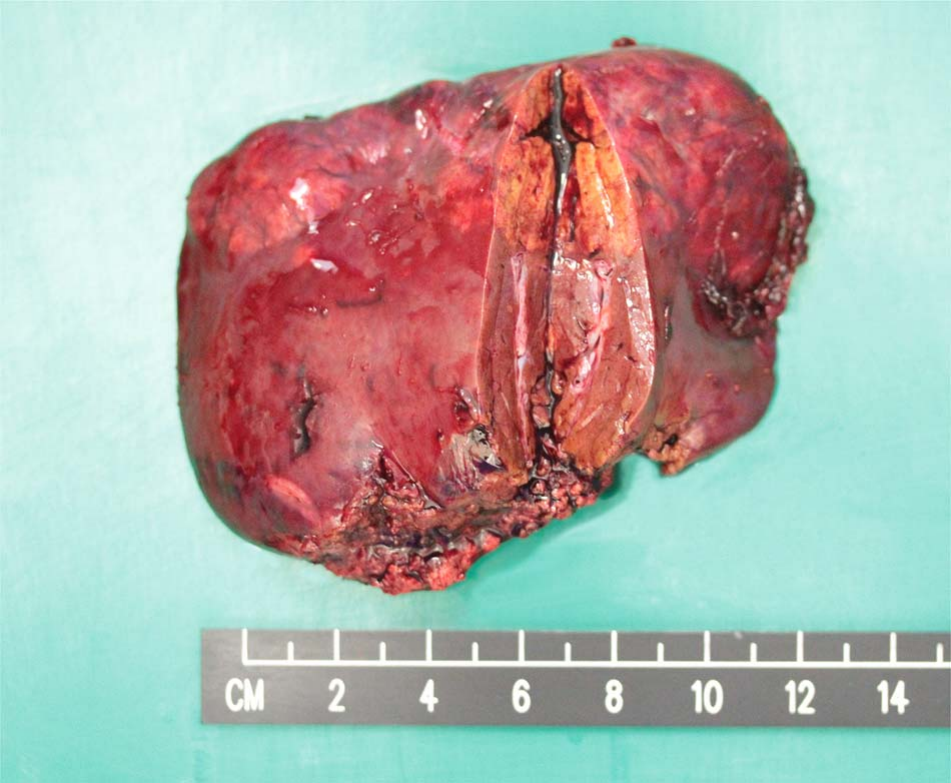
Resected left lateral section showing multiple tan-coloured nodular tumours.

**Figure 3:**
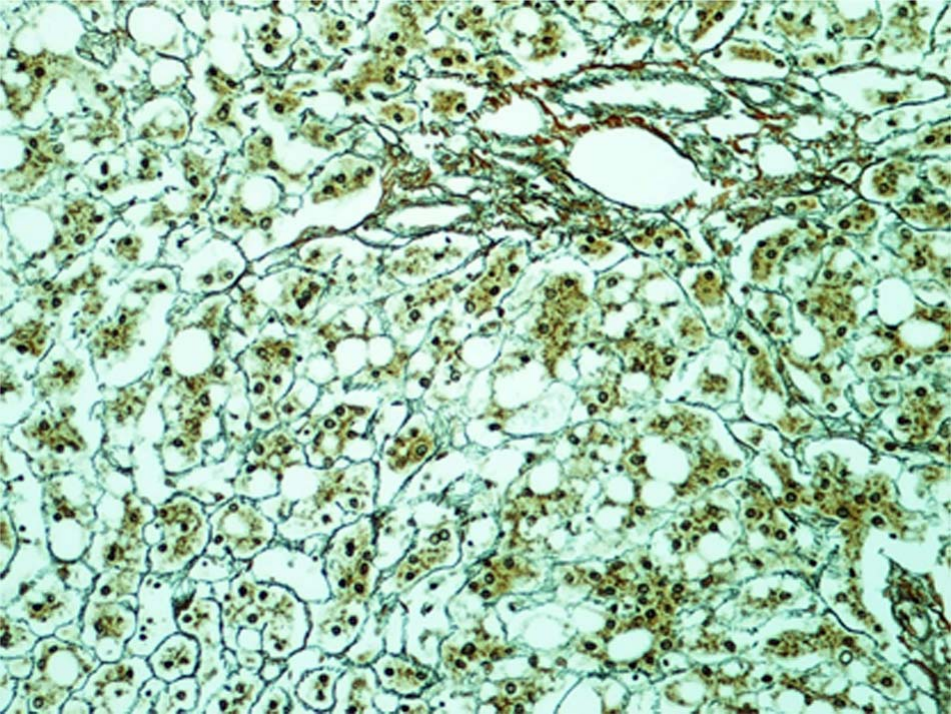
Section of the S2 nodule (magnification 200×). Reticulin stain revealed preserved reticular meshwork in most areas.

**Figure 4:**
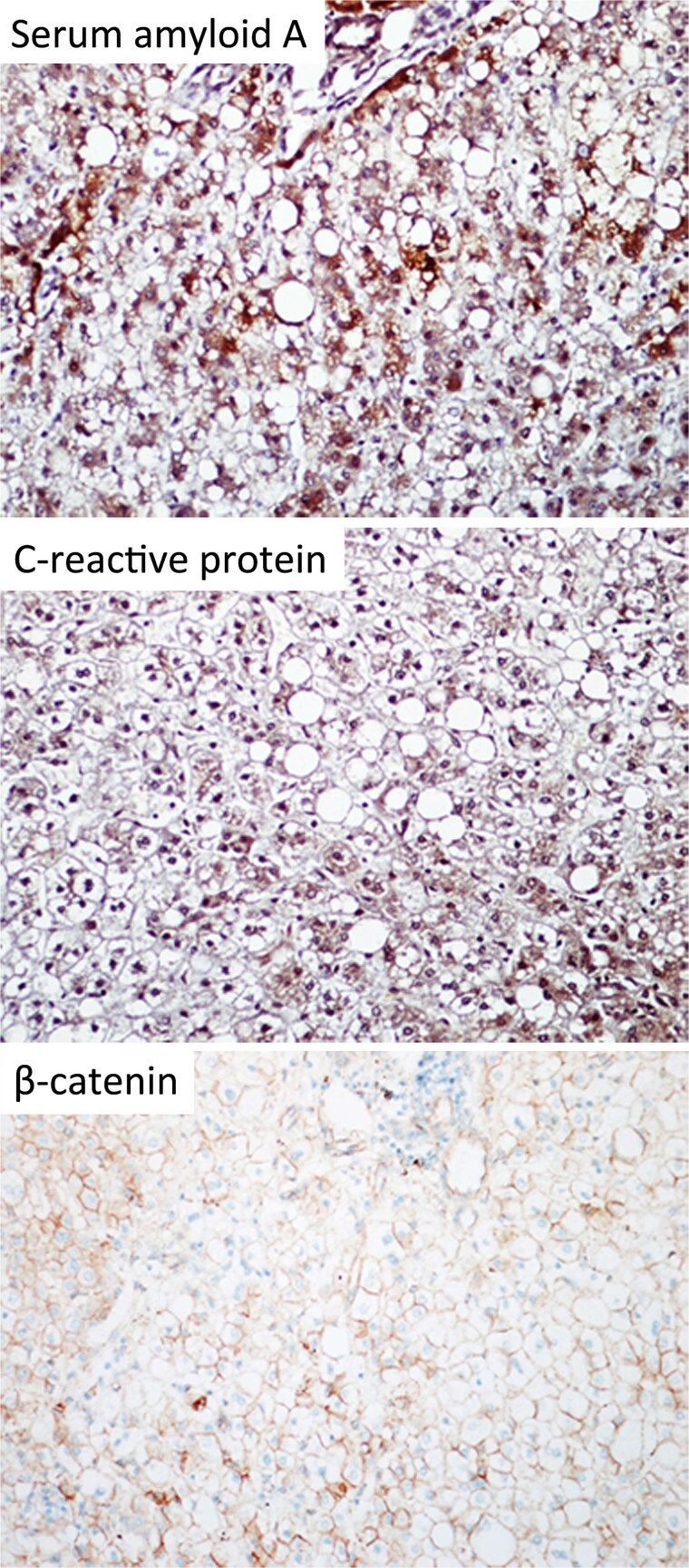
Immunohistochemical staining of the resected specimen (magnification 200×). The tumour cells expressed SAA and CRP. No nuclear expression was observed with β-catenin.

In the view of young age and sizable tumours, surgical treatment was advised to eliminate risk of malignant transformation. Laparoscopic left lateral sectionectomy and wedge resection of segment 4a and segment 8 tumours were performed. The patient had an uneventful operative course. The specimen showed multiple tan-coloured nodular lesions with well-defined border (Fig. 2). The tumour tissues showed severe steatosis. Reticulin stain revealed preserved reticular meshwork in most areas, and the hepatocytes were arranged in up to twin cell plates (Fig. 3). The tumour cells expressed serum amyloid A (SAA) and C-reactive protein (CRP) (Fig. 4). No nuclear expression was observed with β-catenin (Fig. 4). The nontumoral liver tissue showed no fatty change. A diagnosis of inflammatory HCA was confirmed. The overall features are those of multifocal HCA, with the immunoexpression suggestive of inflammatory type.

## Discussion

We report an inflammatory HCA which was metabolically avid for 11C-acetate and 18FDG. The pathological diagnosis was confirmed after surgical resection. This is the first description of 11C-acetate uptake of HCA on PET-CT scan. HCAs are usually evaluated with magnetic resonance imaging (MRI). With Gadolinium contrast, they appear as arterial enhancing lesions which fade in the portal venous and delayed phase. HCAs are classified into six molecular phenotypes: HNF-1α inactivation, β-catenin activating at exon 3, β-catenin activating at exon 7 and 8, inflammatory, sonic hedgehog, and unclassified [3, 4]. For inflammatory HCA, the associated telangiectasia appears as a strong hyper-intensity on T2-weighted MRI [5–7]. The classical radiological features of HCA were absent on the CT scan in our patient. On dual tracer PET-CT scan, the liver tumours were both 11C-acetate and 18FDG-avid. The diagnosis was confirmed to be inflammatory HCA after surgical resection.

PET-CT using 18FDG has been validated for evaluation of various visceral malignancies [8]. However, the sensitivity for detecting primary HCC is limited and is better complemented by 11C-acetate [9]. Acetate is rapidly taken up in cells and converted to acetyl-CoA, which is further utilized in either catabolic or anabolic pathways [10]. In the tumour cells, increased expression of fatty acid synthase (FAS) in the anabolic pathway converts it into fatty acids for tumour growth [11]. 11C-acetate uptake is closely related to increased cellular metabolism. 18FDG avidity has been reported for inflammatory HCA, where the inflammatory infiltrate and the associated cellular activity led to tracer uptake [12]. HNF-1α inactivated HCAs have also been reported with 18FDG uptake [13]. These tumours are associated with significant steatosis. Fat accumulation causes Kupffer cell activation and results in 18FDG uptake [12]. This is in concordance with the findings of 18FDG avidity in focal fatty infiltration [14]. In our case, 11C-acetate avidity can be related to both inflammation and steatosis. In patients with fatty liver disease, cellular FAS expression correlated with the degree of steatosis [15]. Increased FAS activity may have contributed to 11C-acetate uptake in this benign tumour.

Molecular subtyping of HCA allows prognostication and guides treatment decision. β-catenin activating HCA at exon 3 is most frequently associated with malignant transformation. Some molecular subtypes of CHA have distinguishing MRI features. HNF-1α inactivated HCA is associated with extensive steatosis and a diffuse and homogenous signal dropout on T1 weighted images [5]. Inflammatory HCA shows up as marked T2 hyperintensity, which can be diffuse or rim-like at the periphery [6]. When these classical features are absent a biopsy can be considered to determine the molecular subtype for treatment planning. The European Association for the Study of the Liver (EASL) group suggests surgical treatment for HCA >5 cm, but recommends all β-catenin mutated HCA to be resected irrespective of size, due to malignant potential.

In our case, the diagnosis of HCA was confirmed with percutaneous biopsy. However, molecular subtyping was not routinely available in our centre and surgery was contemplated in view of tumour size. Eventually immune-histochemical staining was performed on the resected specimen. The CRP expression and absent β-catenin staining were consistent with inflammatory HCA. In hindsight, availability of molecular subtyping could have added valuable information guiding treatment decision and counselling. Of note, some inflammatory HCA also harbour β-catenin mutation at exon 3 and are at risk of malignant transformation [4]. Immuno-phenotyping may have a role if the decision is made to observe a small radiological inflammatory HCA, to exclude co-existing β-catenin mutation.

In summary, we hereby report a case of multifocal inflammatory HCA with atypical radiological features and 11C-acetate avidity on PET-CT scan. This case illustrates that HCA can also appear as a metabolically active lesion on a dual tracer PET-CT. A percutaneous biopsy might provide valuable information assisting treatment decision when managing a patient with HCA.
